# Analysis of the Mercury Content in Fish for Human Consumption in Poland

**DOI:** 10.3390/toxics11080717

**Published:** 2023-08-21

**Authors:** Barbara Brodziak-Dopierała, Agnieszka Fischer

**Affiliations:** Department of Toxicology and Bioanalysis, Faculty of Pharmaceutical Science, Medical University of Silesia, 30 Ostrogórska Str., 41-200 Sosnowiec, Poland; bbrodziak@sum.edu.pl

**Keywords:** fish, mercury, AAS

## Abstract

Mercury (Hg) is a metal with toxic effects on the environment, including living organisms. Organic Hg derivatives readily penetrate biological membranes and pose a particular health risk. Food of aquatic origin is the main source of human exposure to methylmercury (MeHg). In Poland, the consumption of fishery and aquaculture products has been gradually increasing. The aim of this study was to determine the content of Hg in fish intended for human consumption and purchased in Poland. The total Hg content of the edible parts of different species of marine and freshwater fish was analysed. The range of Hg content in all fish was 0.004–0.827 mg/kg, with an arithmetic mean of 0.084 mg/kg. The concentration of Hg in marine fish (0.100 mg/kg) was higher than in freshwater fish (0.063 mg/kg). The highest concentration of Hg was found in tuna. The Estimated Hazard Quotient (EHQ) calculated for the tuna samples analysed was >1. This may represent a potential health risk for consumers. The consumption of the other fish analysed was considered safe on the basis of the EHQ. The Hg content of the analysed fish samples did not exceed the current limits for food.

## 1. Introduction

Industrial development, agriculture and increasing urbanisation are associated with environmental pollution. This leads to changes in ecosystems and the deterioration of food products of plant and animal origin. One of the most common environmental pollutants is mercury (Hg). The presence of Hg in the environment is partly due to its natural occurrence, but mainly due to anthropogenic activities. Its relatively high persistence in the environment, as well as its mobility and toxic effects, mean that Hg can pose a risk to human health [1,2,3]. An important source of human exposure to mercury is food, mainly of aquatic origin [4,5]. Studies have shown that fish contain much higher levels of mercury than other animal products from the same area [6,7]. In addition to mercury, fish can also be contaminated with other metals (As, Cd, Cr, Pb) [8,9,10].

Fish absorb mercury and heavy metals from the surrounding water as well as from food (plant and animal) [11]. The mercury content in fish depends on a number of factors, such as the area in which they live, the quality of the food, the characteristics of the species and the absorption conditions [12]. The mercury content in fish increases with age, weight and length [13]. Factors that modify the uptake of Hg compounds from water include water pH and dissolved organic carbon content. The highest concentration of Hg in fish muscle is observed when the water pH is around 5 [14]. Poisoning fish with mercury compounds leads to brain damage. It manifests itself as dilation of the gill covers, excessive mucus secretion, increased frequency of respiratory movements, decreased motility, abnormal motor coordination, loss of balance and a lack of appetite [14].

The environmental processes of mercury metabolism are mainly associated with the aquatic ecosystem. Microorganisms produce methylmercury (MeHg), which, as an organic derivative, readily penetrates biological membranes [15]. Mercury accumulates in the trophic chain, especially at the top of the chain in predatory fish [16,17]. In fish tissues, MeHg concentrations can reach up to 90% of the total content of this metal [15,18]. MeHg is mainly deposited in the liver, kidneys and brain of fish and in muscle tissue consumed by humans. In the human body, organic combinations of Hg are particularly toxic, mainly for the nervous system; when combined with cysteine, they readily cross the blood–brain barriers and placenta [19]. The intake of organic mercury by humans depends mainly on the amount of fish consumed and the degree of its contamination [5,7].

The European Union (EU) is one of the world’s most important markets for fish and seafood. Moreover, the consumption of fishery and aquaculture products has been gradually increasing year by year. In 2020, it will amount to 10.41 million tonnes, or about 23 kg per capita. Compared to 2019, consumption increased by 7%. Fish consumption varies considerably between countries (highest in Portugal at 58 kg/person, lowest in the Czech Republic at 6 kg/person) [20]. EU legislation sets a maximum permissible Hg content in fish for human consumption [21,22,23].

The aim of the study was to assess the mercury content in fish for consumption in Poland. Several fish species that are readily available at the point of sale and popular with Polish consumers were analysed. Both the increasing consumption of fishery and aquaculture products and the growing interest in the quality of the food consumed indicate that the study was justified. Typical saltwater and freshwater fish species were tested for Hg content. The rate of exposure to Hg from consumption of these fish and the potential health risks associated with this were estimated. It was determined whether the amount of Hg present in the fish samples tested exceeded acceptable standards. The paper also compares the Hg levels of the different freshwater and saltwater fish species tested and identifies the species with the highest and lowest levels of this metal.

## 2. Materials and Methods

The subjects of the study were fish for human consumption from different points of sale in Poland. Fish were purchased in 2021–2022 at different points of sale all over Poland (hypermarkets, shops selling fish only, and directly at the place of breeding, i.e., breeding tanks). Fish species collection from the market followed a randomised, unbiased sampling strategy. Local pollution variations have not impacted the results. A detailed description of the fish studied is given in Table 1. The study material consisted of samples of the edible parts (flesh) of 68 fish belonging to 18 different saltwater (N = 12) and 6 freshwater (N = 6) species.

The species under consideration in the study were as follows: Atlantic cod (*Gadus morhua*), coalfish (*Pollachius virens*), Atlantic salmon (*Salmo salar*), Yellowfin tuna (*Thunnus albacares*), Atlantic Bluenfin tuna (*Thunnus thynnus*), mackerel (*Scomber scombrus*), Alasca pollock (*Gadus chalcogrammus*), hake (*Merluccius merluccius*), Atlantic halibut (*Hippoglossus hippoglossus*), turbot (*Scophthalmus maximus*), Wels catfish (*Silurus glanis),* Nile perch (*Lates niloticus*), rainbow trout (*Oncorhynchus mykiss*), brown trout (*Salvelinus alpinus*), pike (*Esox lucius*), carp (*Cyprinus carpio*), Pike perch (*Sander lucioperca*) and flounder (*Platichthys flesus*). For some species, common names are used in trade, e.g., the name tuna covers more than a dozen fish species [24]. Product characteristics and data on the origin of the fish were obtained from producers’ descriptions. Fish were both caught and farmed. Free-living marine fish were most frequently sourced from FAO Catch Area 27 (North-East Atlantic), FAO 51 (Western Indian Ocean) and FAO 67 (North-East Pacific) [25].

Marine fish available in Polish shops are mainly delivered as frozen products. They can be found on the market in unaltered form (frozen) as well as with the description “fresh product”, which means that they have been thawed beforehand. Freshwater fish are more often sold as products that have not been frozen. They can also be bought as “live” products, i.e., caught just before being sold (e.g., in the area of aquaculture).

Fish purchased frozen were fully thawed at room temperature prior to testing. Whole small fish (weighing up to 2 kg) were eviscerated (gutted or not), depending on how they were purchased, and then all inedible parts were separated from the edible parts. Edible parts of the whole fish (fish flesh) were taken to prepare test samples, inedible parts were discarded. For large fish, fillets of the edible parts of the fish were used to prepare test samples. In this case, samples of at least 100 g were taken from the central part of the fish [26].

The material to be tested was ground to obtain a homogeneous preparation (Analytical Mill A 11 basic, IKA, Warsaw, Poland). Subsequently, 3 test samples of approximately 100 mg were taken from each preparation (analytical balance RADWAG, Radom, Poland) and sent for analysis.

The Hg content of fish samples was determined by atomic absorption spectrometry (AAS) using an AMA 254 analyser (Altec, Praha, Czech Republic). The following measuring conditions were used: wavelength 253.65 nm, carrier gas–oxygen (O_2_ purity ≥ 99.5%), inlet pressure 200–250 kPa. The measurement technique allows the determination of the total Hg content regardless of its form in the sample. The time of each analytical step was [s]: drying 200 [s], decomposition 250 [s], measurement 90 [s]. The lower detection limit (LOD) is 0.01 ng Hg [27,28]. Every sample was measured 3 times.

Polish certified reference materials (CRMs) for multi-component trace analysis were used to verify the determinations: MODAS-3 herring tissue (M-3 HerTis) and MODAS-5 cod tissue (M-5 CodTis) (MODAS consortium of the Institute of Nuclear Chemistry and Technology, Warsaw, Poland, and the Technical University of Gdańsk, Poland) [29]. Hg content in cod tissue: certified 310 ± 22 ng/g, analysed 272 ± 19 ng/g, recovery 87.7%; in herring tissue: certified 227 ± 21 ng/g, analysed 221 ± 24 ng/g, recovery 97.4%.

The results were statistically analysed using Microsoft Excel and Statistica ver. 13.3 pl (Statsoft, Kraków, Poland). The Hg concentration in the test sample was the arithmetic mean of the 3 measurement results. The Hg content in fish of each species was the arithmetic mean of the metal concentration in all samples of this species. Due to the inconsistency of the obtained results with the normal distribution (Shapiro–Wilk test), the discussion of the results was based on median values, and non-parametric tests were used to compare the statistical variability between the sample groups: the Mann–Whitney U test (for 2 groups) and the Kruskal–Wallis rank ANOVA test (for 3 or more groups). A probability ratio of *p* ≤ 0.05 was considered statistically significant.

An assessment of the health risks that may result from the consumption of fish was made based on the guidelines of the US Environmental Protection Agency (US EPA) [30]. The assessment includes the magnitude, frequency and duration of exposure to the contaminant in combination with the characteristics of the exposed population. The assessed health risk relates to Hg ingested orally and is based on the determination of the average daily dose (ADD) over a lifetime. The ADD is assumed to apply to non-carcinogens and may be applicable to Hg [30].

$$ADD=\frac{C\times CR\times EF\times ED}{BW\times AT}$$

where

C—concentration of contaminant;
CR—average daily contact;
EF—exposure frequency;
ED—exposure duration;
BW—body weight;
AT—averaging time.

Based on the ADD value, the Hazard Quotient (HQ) can be determined:

$$HQ=\frac{ADD}{RfD}$$

where RfD is the reference dose value for Hg = 0.0001 mg/kg/day [30].

The Hazard Quotient is interpreted as follows:

HQ < 1—the risk of adverse health effects in the population is unlikely, and the hazard is negligible;

HQ ≥ 1—the risk of adverse effects in the population is not excluded, and the hazard is probable.

Using the Hg concentrations determined in the test samples, the Estimated Daily Intake (EDI) and the Estimated Hazard Quotient (EHQ) for the fish species tested were calculated from the equations for ADD and HQ, respectively.

The calculations are based on the following assumptions:

C—concentration of Hg in the study probe [mg/g], CR—average daily fish consumption = 32.14 g/day [20], EF—exposure frequency = 365 days, ED = 70 years *, BW = 70 kg,

AT—averaging time (EF × ED = 25,550 days).

* According to statistics, the average life expectancy in Poland is 75.8 years [31]. Regular consumers, i.e., people who consume fishery and aquaculture products at least once a month, are mainly in the 40–54 and over 55 age groups. Young people (15–24 years) consume these products less frequently, both in Poland and on average in the EU [32]. The study assumed ED = 70 years, taking into account infancy and early childhood, when fish consumption is lower than in adults.

## 3. Results

Table 2 shows the statistical analysis of the Hg content of the tested fish samples. The range of Hg content in all fish samples tested (N = 68) was 0.004–0.827 mg/kg, with an arithmetic mean of 0.084 mg/kg and a median of 0.045 mg/kg. The mean Hg content in all saltwater fish samples (0.100 mg/kg) was higher than in freshwater fish (0.063 mg/kg), the median was 0.060 mg/kg and 0.024 mg/kg, respectively. The differences in Hg content between saltwater and freshwater fish samples were not statistically significant (*p* = 0.097).

**Figure 1:**
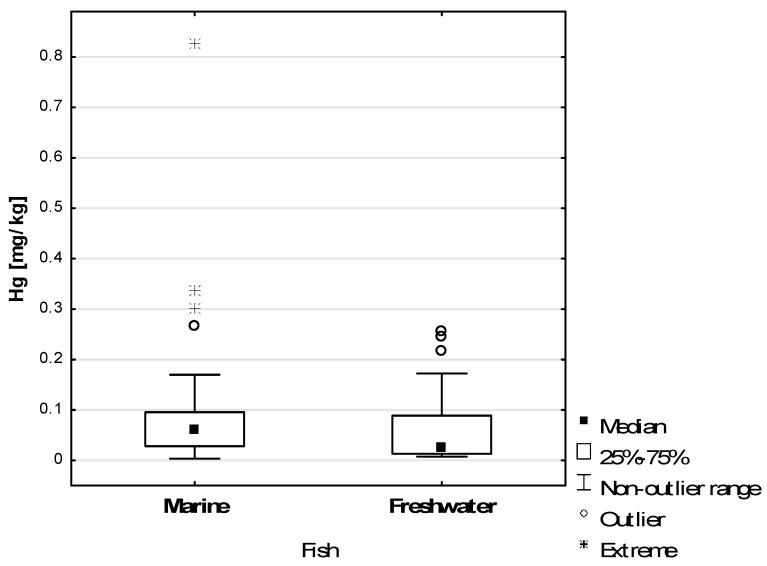
Hg concentration in marine and freshwater fish samples examined.

Figure 1 shows the Hg concentrations in all marine and freshwater fish samples tested. Extreme Hg concentrations above 0.3 mg/kg were recorded in marine species. One sample contained 0.828 mg/kg Hg. The Hg content of freshwater fish samples was less variable—the results obtained tended to fluctuate around the mean value, and no extreme results were recorded.

The tested fish were either captured from fisheries or farmed. The concentration of Hg in all farmed fish (marine and freshwater) was 0.014 mg/kg and was lower than in the captured fish (0.071 mg/kg). Farmed salmon had 0.016 mgHg/kg, and salmon living in the ocean (FAO 61, FAO 67) had 0.018 mgHg/kg. The concentration of Hg in all fish living freely in the ocean was 0.071 mg/kg.

**Figure 2:**
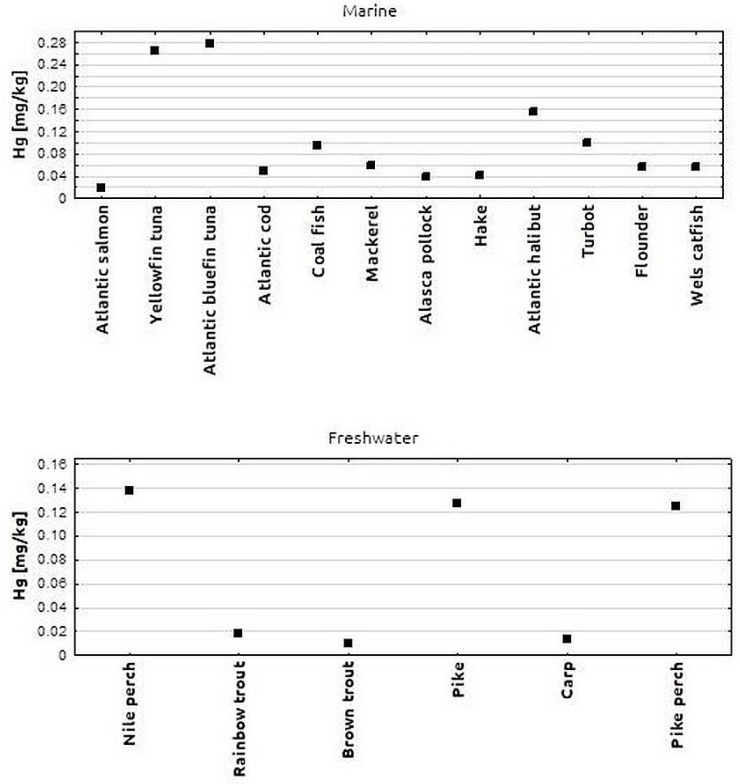
Mean Hg concentration in individual marine and freshwater fish species.

In the next stage of analysis, all fish samples tested were grouped by species. The Hg content of the tested fish species is shown in Figure 2. In marine fish, the highest Hg content (approximately 0.270 mg/kg) was found in tuna and yellowfin tuna. A lower Hg concentration (0.153 mg/kg) was found in halibut samples. The lowest Hg levels among marine fish were found in hake, pollock and salmon samples. In salmon, the Hg concentration was 0.017 mg/kg. The differences in Hg levels between the different marine fish species tested were not statistically significant. The Hg concentrations found in the freshwater fish species examined were distributed as follows: higher Hg concentrations (approx. 0.123 mg/kg) were found in perch, pike and pikeperch, while lower Hg concentrations (approx. 0.01 mg/kg) were found in brown trout, rainbow trout and carp. The differences in Hg levels between all freshwater fish species investigated were not statistically significant.

The EDI and EHQ values calculated for the test samples are presented in Table 3. The EFSA Scientific Panel on Contaminants in the Food Chain (CONTAM) has established a tolerable weekly intake (TWI) of 1.3 µg/kg body weight for methylmercury and 4 µg/kg body weight for inorganic mercury [21]. The daily intake is 0.186 µg/kg BW for MeHg and 0.571 µg/kg BW for inorganic Hg. To ensure that the above standards are not exceeded, the EDI values should not exceed 0.000186 mg/kg BW (for exposure to MeHg) and 0.000571 mg/kg BW (for exposure to inorganic Hg), taking into account the study population and the concentration of Hg in fish. For our results, the EDI values were 0.000004–0.000128 mg/kg body weight. They were highest for saltwater fish samples of tuna (0.000125 mg/kg b.w.) and yellowfin tuna (0.000122 mg/kg BW). In none of the fish species tested did the results exceed the recommended standards for MeHg and inorganic Hg [18]. The EHQ values for the fish samples tested ranged from 0.04 to 1.28 (Table 3).

**Figure 3:**
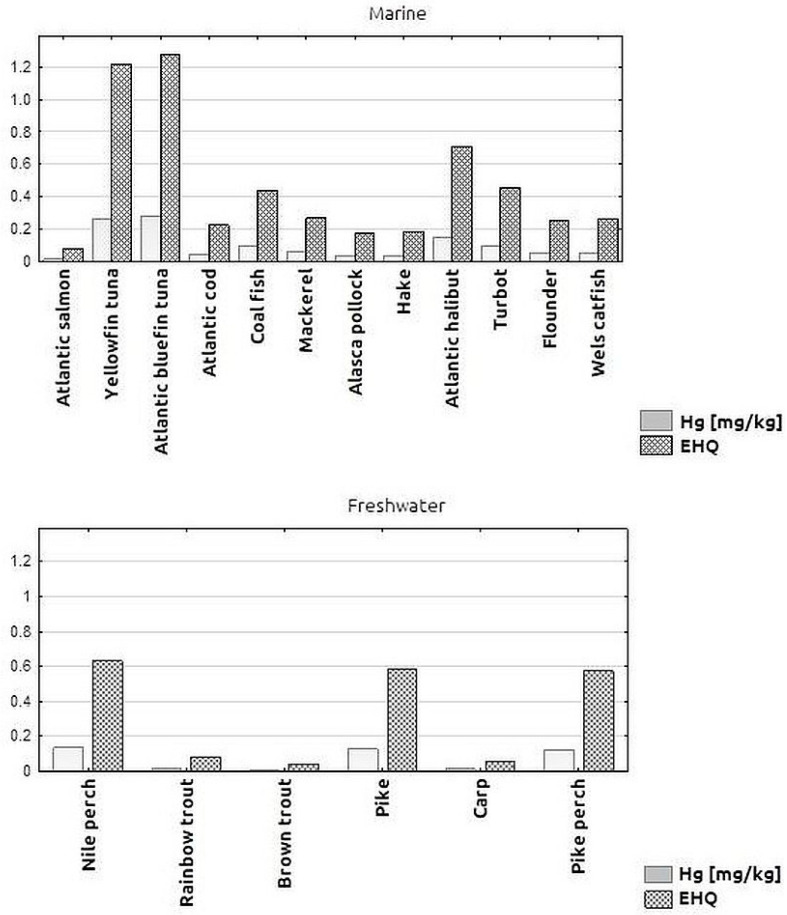
Mean Hg concentration [mg/kg] in individual marine and freshwater fish species and Estimated Hazard Quotient (EHQ) values.

Figure 3 shows the Hg concentrations in the samples of different marine and freshwater fish species and the corresponding calculated EHQ values. For freshwater fish, the EHQ values ranged from 0.04 to 0.63, with none of the cases analysed exceeding the value of 1.00, which would indicate a potential health risk. For marine fish species, the EHQ values ranged from 0.07 to 1.28, with the EHQ for yellowfin tuna and tuna species exceeding 1. For the other marine fish samples tested, the calculated EHQ value was lower and did not exceed 1.00, e.g., for halibut, EHQ = 0.71; for turbot and saithe, around 0.4. The lowest health risk, expressed by the lowest EHQ value, was found for salmon (EHQ < 0.1), followed by pollock and hake (EHQ < 0.2).

## 4. Discussion

Fish is an essential part of a balanced human diet. They are a source of healthy protein, polyunsaturated fatty acids, minerals and vitamins [33]. The share of fishery and aquaculture products in the Polish diet is gradually increasing. A 2% increase in consumption to about 13 kg per person per year has been reported for 2019–2020 [32,34]. The most commonly consumed fish species by Poles are pollock, herring, panga and salmon. Together, these species account for 56% of fish consumption. The main types of marine fish consumed are herring, pollock, sprat, mackerel, horse mackerel and cod [32]. There has also been a local increase in fish catches in recent years, mainly freshwater fish [34]. The consumption of freshwater fish will increase by more than 9% between 2010 and 2020. Poles consume mainly frozen products, and their consumption is higher than the EU average [32].

In addition to its nutritional value, fish flesh can be a source of contaminants, including Hg. Acceptable levels of contaminants in food are set by regulation, and the maximum level of Hg in most species of fish and fish products is 0.5 mg/kg [22,23]. In all the fish samples analysed, the Hg content ranged from 0.004 to 0.827 mg/kg. An extremely high Hg content (0.827 mg/kg), different from the others, was found in only one sample, a fish of the tuna species (*Thunnus thynnus*), which, according to the manufacturer’s description, came from FAO fishing area 34. In the study by Nicklisch et al. [35], in yellowfin tuna caught from 12 different locations worldwide, methylmercury levels were in the range of 0.03 to 0.82 mg/g wet weight. Mean mercury levels were only weakly associated with fish size or lipid content. The geographic area of origin of fish can affect mercury levels [35]. The study shows that Hg levels in marine fish vary depending on where they are caught. Tuna species (*Thunnus alalunga* and *T. albacares*) from African waters had higher concentrations of heavy metals (Hg, As and Pb) in muscle than samples of the same species caught in other areas [36]. A study by Barone et al. [37] found Hg concentrations of 0.74 mg/kg in tuna samples. An even higher concentration of Hg in tuna (1.7 mg/kg) was found in a study by Annibaldi et al. [38]. Tuna are a group of predatory, large and long-lived marine fish characterised by a particular ability to accumulate Hg [38]. The limit for Hg in carnivorous fish is 1.0 mg/kg, twice that of other species [23]. This limit was not exceeded in our tuna sample with the highest Hg content (0.827 mg/kg). In contrast, the mean Hg concentration of all tuna samples tested together did not exceed 0.3 mg/kg. Thus, both the tuna samples and all fish samples tested did not exceed acceptable standards [22,23].

Portuguese research (Portugal is one of the countries with the highest fish consumption per capita in the world and the highest at EU level) ranked Hg concentration in fish species as follows: hake > horse mackerel > codfish > octopus [39]. In the case of mackerel, a very popular fish among Polish consumers, the Hg concentration tested was many times lower than the permissible limit, at 0.06 mg/kg. The results obtained were similar to those obtained in the study by Bilandžić et al. (0.08 mgHg/kg) [40].

Similar to our study, Barone et al. [37] carried out a study on fish as a food product. In various 11 common species of marine fish purchased in shops in Italy, the average Hg content was found to be 0.40 mg/kg. This value was several times higher than that found in our marine fish samples (0.10 mgHg/kg). It should be noted that the studies cited [37] were carried out earlier than ours. Long-term studies of Hg levels in fish clearly show a decreasing trend. In this respect, extensive studies have been carried out by Julshamn et al. [41,42] on the species Greenland halibut (*Reinhardtius hippoglossoides*). Fish samples tested in 2006 contained 0.03–1.1 mgHg/kg [41], some exceeding the normative values. As reported in a study by Bank et al. [18], the bioaccumulation of Hg in halibut flesh decreased by approximately 35–50% from 2006 to 2015. This result was shown to be the result of both reduced environmental Hg emissions and a change in the trophic position of the fish and its diet. The form of Hg found in these fish was mainly MeHg (>77%), which has a high bioavailability [18]. In our samples of halibut purchased in shops in Poland, the concentration of Hg was 0.152 mg/kg, which does not exceed the level defined as safe for consumers. However, in their study, Julshamn et al. [42] pointed out that, in light of the other metals they studied (Pb, Cd, As) found in fish, only the Hg content may be of concern to consumers and should be monitored [42]. Our estimated EHQ values showed that a potential health risk to consumers may occur with the consumption of only two of the fish species tested: tuna and yellowfin tuna (EHQ > 1). For the remaining fish species tested, the health risk was considered unlikely, and the risk to the consumer population was considered negligible (EHQ < 1). The health risk factor did not exceed a value of 1 (with values ranging from 0.04 to 0.74) for the different species of edible fish investigated by Barone et al. [33], and the same was true for the study by Łuczyńska et al. [43,44]. The highest EHQ values, as in our study, were obtained for Atlantic bluefin tuna (0.74), as well as for swordfish, European conger eel and rosefish [37]. The aforementioned study by Julshamn et al. [42] on the content of various elements in halibut meat showed that Hg is closely correlated with the content of other heavy metals. Even low concentrations of Hg can be positively correlated with arsenic (As) concentrations. Based on the results, the researchers recommend that consumers control the amount of fish they eat and not exceed two meals of Greenland halibut per week for health and safety reasons [42].

Selenium (Se) has a natural antagonistic effect on mercury in fish. Research by Belmonte et al. [45] on Atlantic bluefin tuna showed that kidneys were the tissue with the highest concentrations of Hg and Se. The Selenium Health Benefit Value (HBVSe) was positive in every sample and tissue, indicating that Se plays an important role against mercury, not only in the muscles [45].

The migration of freshwater fish is more restricted than that of marine fish. High concentrations in fish were closely related to concentrations in the rearing environment [46]. Stężycka et al. [47] analysed the Hg content in fish caught in the Vistula River (near Warsaw and Włocławek) in Poland in the years 1998–2002. The average Hg content of the fish examined increased in successive years and was as follows: 1998–0.122 mg/kg, 2000–0.169 mg/kg and 2002–0.209 mg/kg. The results obtained in 2021–2022 were many times higher than ours, where an average of 0.063 mgHg/kg was found in the edible parts of freshwater fish. In the same study [47], the highest Hg content was found in the predatory fish pike (0.25 mg/kg). A similarly high level of Hg (0.297 mg/kg) was found in pike from north-eastern Poland (Warmia and Mazury regions) [48]. A similar trend of changes in Hg levels in fish flesh, depending on the species, has been shown by other studies from Poland [44,49]. Relating these results to ours, the Hg content in pike was lower at 0.128 mg/kg. However, both pike and another predatory fish species, perch, were characterised by the highest Hg concentrations among the freshwater fish species we analysed.

Łuczyńska et al. [44] found Hg levels in fish purchased in shops in Poland in the range of 0.006–0.138 mg/kg. In contrast to our study, freshwater fish had higher Hg concentrations than marine fish. However, similar to our study, the differences in Hg content in the flesh of freshwater and marine fish did not show statistical significance. Among freshwater fish, the highest Hg content was found in perch (0.016 mg/kg), followed by rainbow trout (0.015 mg/kg) and the lowest in carp (0.006 mg/kg) [44]. In our study, the obtained Hg levels in fish were higher, but in analogous species they decreased in the same order: perch (0.138 mg/kg), rainbow trout (0.018 mg/kg) and carp (0.013 mg/kg). A similar trend, with higher Hg levels in perch than in carp, was shown in the study by Leśniewska et al. [50]. In this study, the Hg concentration in carp was 0.156 mg/kg [50], which is comparable to the study by Has-Schon et al. [51] and many times higher than in others [49,52] and in our study (0.013 mg/kg). Commercially available carp in Poland are farmed fish. In addition to environmental conditions, the Hg content in the flesh of fish of this species may be influenced by the quality of the feed used for feeding. This is suggested by the study of Kenšová et al. [53], who determined the dynamics of changes in the Hg content of rainbow trout at different life stages by analysing feed samples. The lowest Hg concentrations were found in 14-day-old embryos (eggs) and the highest in 18-month-old individuals (0.128 ± 0.048 mg/kg). The amount of Hg in the diet ranged from 0.0126 to 0.0859 mg/kg. A significant correlation was found between Hg intake and fish tissue Hg levels (*p* < 0.001) [53].

A fish species that, like carp, is mainly farmed in Poland is trout. In rainbow trout from fish farms located in different regions of Poland, the average Hg content was 0.025 mg/kg [54], a result very close to ours (0.020 mgHg/kg). In contrast, in the study by Ćwieląg-Drabek et al. [55], the Hg content in trout was about twice as high, at 0.058 mg/kg. In fish reared under controlled conditions, i.e., from organic fisheries in Poland, the Hg content was in the range of 0.008–0.056 mg/kg, the highest being in sturgeon [56]. On the other hand, in fish of different species (carp, roach, perch, pike) from a water body affected by pollution from a landfill in Lithuania, the range of Hg content was 0.02–0.5 mg/kg [57]. In this study, it was observed that bentophagous fish feeding mainly in bottom areas (carp, roach) accumulated less Hg than predatory fish (perch, pike). The mean Hg concentration in the muscles of benthic fish (0.12 mg/kg) was twice as low as in predatory fish (0.25 mg/kg) [57]. Similarly, in our study, the Hg concentration in carp was many times lower than in perch.

According to a study in Italy, Hg levels in catfish from the river Po ranged from 0.51 to 1.43 mg/kg, and the acceptable standard was exceeded in 18% of the fish samples tested. The highest mercury levels were found in muscle and the liver [58]. These results are higher than those from Poland, where both in our study and in others [6,44,47,48,54,55,56], the permissible level of Hg in fish flesh was not exceeded. Fish from the Vestonice natural water reservoir in the Czech Republic were also found to have Hg levels lower than the permissible standards (0.02–0.46 mg/kg) [59]. Research by Kenšová et al. [59] involved fish collected from drinking water sources and was published in 2005. Mercury content in these studies (0.02–0.46 mg/kg) was higher than in fish studied by Łuczyńska et al. [44]. Fish in the research of Łuczyńska et al. [44] were, as in our study, purchased from markets. They covered an earlier period than ours and were published in 2017. Higher mercury content in Kenšová et al.’s study [59] compared to Łuczyńska et al.’s study [44] may result in different times of research conducted—2005 vs. 2017. The decrease in mercury concentrations in fish in the following years was also confirmed in the study of Bank et al. [18], which involved Greenland halibut and was conducted during the years 2006–2015. As in our and other studies [47,48,49], the highest Hg concentrations were found in predatory fish. In a study by Kenšová et al. 2010 [59], differences between Hg concentrations in carp and predatory fish species (asp, pike, pikeperch) were statistically significant (*p* < 0.01). Such significance was not found in our study.

Fish from the Danube, which flows through Croatia, have been found to contain between 0.191 mgHg/kg (omnivorous species) and 0.441 mgHg/kg (plankton eaters) [13]. Fish from the same river in Serbia had slightly lower Hg levels (0.140 to 0.327 mg/kg) [60]. The amount of Hg increased with the total length and weight of the fish [13]. In the flesh of fish caught in the Nitra River in Slovakia, Hg concentrations ranged from 0.34 to 3.64 mg/kg, with an average of 0.88 mg/kg. The highest Hg content was found in catfish (1.53 mg/kg). It was concluded that the consumption of the fish tested could pose a risk to human health, as the permissible standards were repeatedly exceeded [61].

A study of fish from three rivers in South America (Bolivia) was presented by Pouilly et al. [62]. The Hg content of predatory fish (0.105–0.151 mg/kg) was higher than that of herbivorous fish (0.039–0.052 mg/kg) [62]. In contrast, in fish from African lakes (Burkina Faso) characterised by a short food chain length (giraffe catfish, Senegalese longfin mako, Nile tilapia, Nile perch), the Hg content was in the range of 0.006–0.230 mg/kg [63].

Food from aquaculture and fisheries is a recognised source of consumer exposure to Hg. However, other food ingredients may also be contaminated with this metal [64,65]. The results of a study by Yan M. et al. [65] on the heavy metal content of milk and dairy products and the health risks associated with their consumption showed that in the products tested, which are available on the Chinese market, the hazard index for the 11 metal elements analysed was less than 1, indicating negligible non-carcinogenic health risks [65]. Body exposure results from the consumption of various Hg-contaminated products. In the case of aquatic food, the main form of Hg is organic compounds, which are more toxic to living organisms than its other forms. In addition, studies have shown that Hg in fish may pose additional health risks by interacting with other heavy metals [42]. Consumers are advised to be cautious and to limit the consumption of fish, especially predatory fish, from waters with high levels of heavy metals [42]. These recommendations apply to particularly sensitive groups of consumers. In Poland, the consumption of tuna and king mackerel, among others, is not recommended for pregnant and lactating women and children [66]. Research results highlight the importance of continuous monitoring of total mercury and MeHg levels in fish, especially predatory fish, to avoid the risk of overexposure in the population [15].

## 5. Conclusions

The range of Hg content in all fish samples tested was 0.004–0.827 mg/kg. The mean Hg content in marine fish was higher (0.100 mg/kg) than in freshwater fish (0.063 mg/kg). The differences in Hg levels between freshwater and marine fish were not statistically significant.

Among marine fish species, tuna had the highest Hg content. Samples of hake, pollock and salmon had the lowest Hg levels.

Hg concentrations in freshwater fish varied as follows: higher Hg concentrations were found in perch, pike and pikeperch, and lower concentrations in trout and carp.

In all fish samples tested, the results did not exceed the recommended content standards for methylmercury and inorganic mercury [18].

The calculated EHQ indicates that the consumption of marine fish belonging to the tested tuna and yellowfin tuna species may pose a potential health risk.

The consumption of other fish species than tuna included in the study has not been associated with a health risk to the population in the large studies that have been conducted.

Research results point to the importance of monitoring mercury levels in fish for consumption. Consumers, especially risk groups (pregnant and lactating women and children), should be advised to consume fish species with low Hg contents and control or avoid the amount of the ones with high contents.

## Figures and Tables

**Table 1:** Description of the fish investigated.

Fish	Species	Latin Name	Number of Fish	Origin/FAO Catch Area	Origin—Supplementary Information
Marine	Atlantic cod	*Gadus morhua*	9	FAO 27	Norwegian Sea
	Coal fish	*Pollachius virens*	1	FAO 27	North Sea
	Atlantic salmon	*Salmo salar*	1	FAO 61, FAO 67	China
			2	farmed	Norway
	Yellowfin tuna	*Thunnus albacares*	1	FAO 51	—
	Atlantic bluefin tuna	*Thunnus thynnus*	6	FAO 34	—
	Mackerel	*Scomber scombrus*	1	FAO 27	North Sea
	Alasca pollock	*Gadus chalcogrammus*	5	FAO 61, FAO 67	Pacific Ocean
	Hake	*Merluccius merluccius*	4	FAO 41	—
	Atlantic halibut	*Hippoglossus hippoglossus*	4	FAO 27, FAO 21	—
	Turbot	*Scophthalmus maximus*	1	FAO 27	—
	Flounder	*Platichthys flesus*	2	FAO 27	—
	Wels catfish	*-Silurus glanis*	1	—	—
Freshwater	Nile perch	*Lates niloticus*	5	—	—
	Rainbow trout	*Oncorhynchus mykiss*	1	—	—
	Brown trout	*Salmo truta*	1	—	—
	Pike	*Esox lucius*	3	—	—
	Carp	*Cyprinus carpio*	8	farmed	Poland
	Pike perch	*Sander lucioperca*	5	—	—

—no data.

**Table 2:** Statistical analysis of Hg content in the investigated fish samples [mg/kg].

Fish	N	AM ± SD	Median	Quartile		*p*
				Q_1_	Q_3_	
All	68	0.084 ± 0.120	0.045	0.017	0.092	
Marine	38	0.100 ± 0.146	0.060	0.028	0.096	0.097
Freshwater	30	0.063 ± 0.073	0.024	0.013	0.089	

AM—arithmetic mean, SD—standard deviation, Q_1_—first quartile, Q_3_—third quartile, *p*—probability ratio.

**Table 3:** Hg concentration (C) in the studied marine and freshwater fish species and Estimated Daily Intake (EDI) and Estimated Hazard Quotient (EHQ) values.

Type	Species	C [mg/kg]	EDI [mg/kg Body Weight]	EHQ
Marine	Atlantic salmon	0.017	0.000008	0.077
	Yellowfin tuna	0.265	0.000122	1.217
	Atlantic bluefin tuna	0.278	0.000128	1.277
	Coal fish	0.096	0.000044	0.439
	Atlantic cod	0.049	0.000022	0.224
	Mackerel	0.058	0.000027	0.268
	Alasca pollock	0.037	0.000017	0.171
	Hake	0.041	0.000019	0.187
	Atlantic halibut	0.154	0.000071	0.707
	Turbot	0.099	0.000045	0.454
	Flounder	0.056	0.000026	0.255
	Wels catfish	0.058	0.000026	0.263
Freshwater	Nile perch	0.138	0.000063	0.632
	Rainbow trout	0.018	0.000008	0.084
	Brown trout	0.010	0.000004	0.043
	Pike	0.128	0.000059	0.586
	Carp	0.013	0.000006	0.061
	Pike perch	0.125	0.000058	0.575

## Data Availability

Not applicable.
